# Larger Fish Have Larger Brains With More Neurons Across but Not Within Cohorts Raised in Different Growth Conditions

**DOI:** 10.1002/cne.70090

**Published:** 2025-09-25

**Authors:** Magda C. Teles, Gonçalo M. Melo, Suzana Herculano‐Houzel, Rui F. Oliveira

**Affiliations:** ^1^ Gulbenkian Institute for Molecular Medicine Oeiras Portugal; ^2^ Department of Psychology, Vanderbilt Brain Institute Vanderbilt University Nashville Tennessee USA; ^3^ ISPA ‐ Instituto Universitário Lisbon Portugal

**Keywords:** brain evolution, brain size, individual variation, numbers of neurons, phenotypic plasticity

## Abstract

Comparative work on brain size variation across vertebrates has shown that larger species have larger brains and that larger brains have more neurons across species in each clade. This trend supports the expectation that larger bodies require larger brains with more neurons but is at odds with the finding that within a species, larger animals do not necessarily have larger brains, and larger brains do not have more neurons. While the latter finding is inconsistent with the expectation that larger brained species evolve through selection of larger brained individuals, the lack of correlation between brain size and numbers of neurons across individuals of a same species might be due to the small range of variation that is typically found within a species. Here, we take advantage of ecologically regulated indeterminate growth exhibited by the cichlid fish tilapia (*Oreochromis mossambicus*) raised under different population densities to generate an over 30‐fold variation in body mass across adult individuals of the same age. We find that across the cohorts of individuals raised with different growth opportunities provided by different population densities, larger animals have larger brains with more neurons that occur at similar neuronal densities, as applies to interspecific scaling in several vertebrate clades. Within each cohort raised at a given population density, however, those animals with more neurons have higher neuronal densities, but not larger brains or bodies, though the latter scale together—as applies to intraspecific scaling in mice and chickens. We conclude that brain size and number of neurons are determined independently across individuals in a population but scale together across cohorts, in step changes that accompany varying opportunities for growth, in the absence of any selection pressure. Based on these results, we propose a model of brain evolution through plastic changes in response to changing environmental opportunities that accounts for intra‐, inter‐, and clade‐specific patterns of brain scaling and diversity.

## Graphical abstract

Teles et al. propose a model of brain evolution that explains how come fish with larger bodies have larger brains with more neurons across cohorts raised in different growth conditions, while individuals within a cohort do not, mimicking the different findings across and within species, respectively.

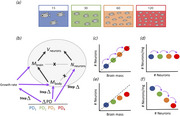

## 1 Introduction

It is often assumed that larger animals have larger brains and that larger brains have more neurons, both within and across species. Indeed, comparative work across species on brain size variation has confirmed the occurrence of positive correlations among these variables across over 200 species of amniotes within their clades (Herculano‐Houzel et al. 2007, 2014; Olkowicz et al. 2016; Kverková et al. 2022). The trend appears to be supportive of the assumption that larger bodies require larger brains with more neurons (Jerison 1973). This assumption is a critical one for evolutionary neuroscience because of the implication that, if neurons are the signal processing units of brains (Herculano‐Houzel 2017) and larger individuals have larger brains with more neurons, it follows that individuals with larger brains, once body size is discounted, would have a cognitive advantage over smaller ones (Jerison 1973). In line with this scenario, a study on artificial selection for brain size in guppies found that female individuals selected for larger residual brain mass (after accounting for body mass) also have a larger overall number of neurons (Marhounová et al. 2019). Thus, the evolution of species with larger brains and enhanced cognitive abilities could result from natural selection acting on preexisting intraspecific variation for brain size, through positive selection of the larger animals with the largest brains and the most neurons. In this scenario of evolutionary diversity in brain size that simply expands on individual diversity, the scaling relationships that apply to body size, brain size, and number of neurons across species must be a simple extension of the allometric relationships across individuals of the same species.

However, the available data on intraspecific variation in brain size and neuron numbers show that the relationships between body size, brain size, and number of neurons that hold across species are not an extension of the allometric relationships across individuals of the same species. Across rodent species, larger animals have larger brains with more neurons that occur at lower densities (Herculano‐Houzel et al. 2007, 2011), but across similar‐aged individuals of a noninbred strain of laboratory mouse, no significant correlation was found between body size, brain size, and number of neurons (Herculano‐Houzel et al. 2015). Instead, there was a strong *positive* correlation between numbers of neurons and neuron density in every brain structure, whereas these correlations are strongly *negative* across species of rodents (Herculano‐Houzel et al. 2015). While that study examined a relatively small cohort of 19 individuals, similar results were reported recently across a much larger cohort of 66 intercrossed domestic and junglefowl of both sexes, showing a similar twofold variation in body size (Cunha et al. 2022). The question thus remains of whether the effects of selection on brain size, be it natural or artificial, as performed in guppies (Kotrschal et al. 2013), occur by expanding a pattern of scaling of larger brains with more neurons that already exists in natural populations. This could happen, for example, by increasing the offspring of some individuals with larger brains within a cohort, or through other mechanisms, such as phenotypic plasticity in response to environmental changes, whether intentional or inadvertent. Intraspecific variation in brain size has already been shown to respond to factors such as predation pressure (Gonda et al. 2009, Gonda et al. 2011), environmental complexity (Fisher et al. 2015; Triki et al. 2020; Reyes et al. 2022), and resource availability (Roth and Pravasudov 2009).

Here, we use an experimental approach that capitalizes on phenotypic plasticity to examine intraspecific variation in brain size and number of neurons within a single generation, which mimics the variation that occurs across natural environments, but with the advantage that we can rule out any role of selection in the outcomes. We take advantage of the fact that fish have continued adult neurogenesis (Zupanc 2021) as well as indeterminate body growth in some species, which may be socially regulated, to create a large range of body sizes of both sexes to ask the question of whether larger individuals consistently have larger brains with more neurons. We used the Mozambique tilapia (*Oreochromis mossambicu*s), an African cichlid fish for which a detailed brain atlas is available and adult neurogenesis has been characterized in detail (Simões et al. 2012; Teles et al. 2012), and whose body growth is reliably regulated by the social environment (Oliveira 1995). We used fish that hatched around the same day and raised them in different population densities (15, 30, 60, or 120 individuals) with ad libitum food in four cohorts in tanks of the same volume, but each at a different population density, for two different periods of time (5 or 12 months). We then used the isotropic fractionator method (Herculano‐Houzel and Lent 2005) to determine the cellular composition of the different main divisions of the brain of these individuals, and applied least‐square fitting of linear regressions to the log‐transformed data to assess the scaling rules between body size, brain size, and neuron numbers, both within each cohort (individuals raised in one same tank) and across the four population densities (individuals raised in different tanks). Our approach thus maximized the chances for finding, across individuals of the same species, the positive correlations across body size, brain size, and number of neurons, together with the negative or null, but not positive, correlation between number of neurons and neuronal density predicted by the hypothesis of evolution of larger brains through natural selection of the largest individuals within a cohort.

## 2 Methods

### 2.1 Animal Housing and Ethics

A total of 80 Mozambique tilapia, *O. mossambicus* (Teleostei, Cichlidae), from a stock bred in our laboratory at ISPA were used in this study. All fish were maintained in four identical glass tanks (50 cm length × 25 cm width × 30 cm height) with a fine gravel substrate. Each tank was supplied with a double filtering system (gravel and external biofilter) and continuous aeration. Water quality was monitored twice per month for nitrites (0.2–0.5 ppm), ammonia (<0.5 ppm, Pallintest kit), and pH (6.0–6.2). Fish were kept at a temperature of 26°C ± 2°C, under a 12 Light:12 Dark photoperiod, and fed ad libitum with small commercial cichlid floating pellets.

### 2.2 Experimental Design

To create differences in body size, four experimental groups with different population densities were set, with 15, 30, 60, and 120 fish, respectively. All fish hatched around the same day (1–2 days difference) and were intermixed from four different clutches into the four identical tanks. The groups were formed 2 days after hatching at the latest. After the groups were set, the remaining individuals of the same clutches were maintained in a single tank (the “sibling tank”) with 150–180 individuals under regular housing conditions for 5 months. Because we wanted to depict a full range of body sizes, two collection points were performed: one at 5 months, when the animals were young adults, and a second one at 12 months, to investigate if the scaling rules that applied to early adulthood were maintained during continuum growth, that is, if there was a continuum across all body sizes. Ten fish were collected at each time point. After the 5‐month sampling, population densities in the tanks housing 60 or 120 fish were restored by moving 10 fish from the sibling tank to each tank. The missing 10 fish from the tank housing 15 fish were replaced from two sources: the six largest fish from the sibling tank, and the four largest fish from the tank housing 30 fish. Finally, the missing 14 fish from the tank that housed 30 fish were replaced with siblings from the sibling tank. Animals were then allowed to grow for another 7 months until the second sampling at age 12 months.

### 2.3 Tissue Collection

**FIGURE 1:**
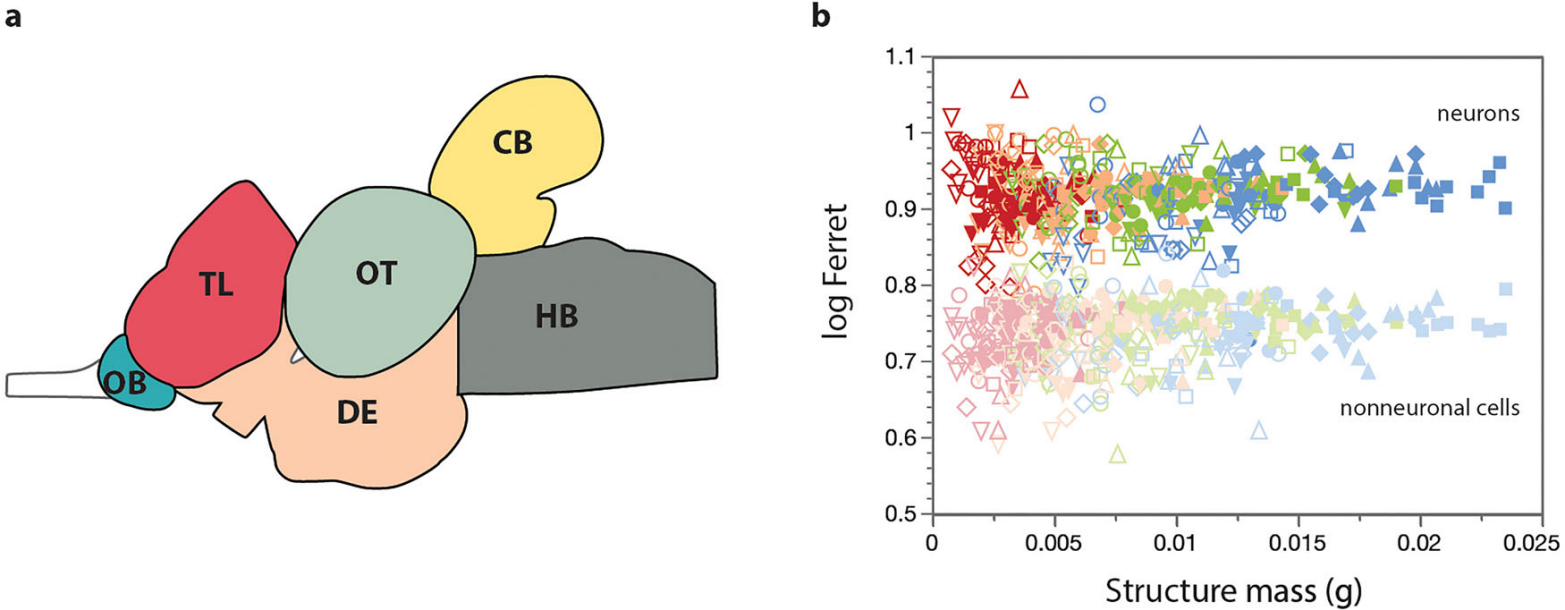
Tissue collection and analysis. (a) Schematic of the microdissection of the tilapia brain showing the brain areas collected: olfactory bulbs (OB), telencephalon (TL), diencephalon and midbrain (DE), optic tectum (OT), cerebellum (CB), and hindbrain (HB). (b) Values of log Feret, which indicate the diameter of the nuclei, in cell nuclei classified as neuronal (colors) and nonneuronal (grayed out) show that these are two clearly distinct groups. All datapoints are shown, for the different brain structures (TL, circles; DE, squares; OT, upright triangles; CB, downturned triangles; HB, lozenges). Colors in (b) represent the different population densities, as in the main figures (blue, 15 fish/tank; green, 30 fish/tank; orange, 60 fish/tank; red, 120 fish/tank). Unfilled symbols, 5‐month‐old animals; filled symbols, 12‐month‐old animals.

For sample collection, 10 fish from each population density (15, 30, 60, and 120 fish) were randomly selected. Fish were sacrificed with an overdose of tricaine solution (MS222, Pharmaq; 1200 mg/L), and the brain was separated into six major brain areas under a stereoscope (SZ‐PT Olympus): olfactory bulbs (OB), telencephalon (TL), diencephalon + midbrain (DE), optic tectum (OT), cerebellum (CB), and hindbrain (HB) (Figure 1a). To minimize variation in tissue collection, all dissections were performed by the same experienced researcher (M.C.T.). Biometric data were also collected: total length (from the tip of the snout to the end of the caudal fin), standard length (from the tip of the snout to the end of the caudal peduncle), and body weight (g). Postmortem gonadal inspection was performed to confirm the sex of each individual. After dissection, the brain regions were placed in paraformaldehyde (4% solution in a 0.1 M phosphate buffer) for 24 h and subsequently weighed on an analytical scale with 0.0001 g sensitivity. The samples from the olfactory bulbs were removed from the analysis, as due to their very small size, we considered that the measurements of the mass of the structure were not reliable. Samples were stored in PBS (x1) for immediate use or placed in an antifreeze solution (30% glycerol and 30% ethylene glycol in PBS at a concentration of 0.024 M) for long‐term storage at −20°C.

### 2.4 Isotropic Fractionator

For the analysis of the cellular components in the different brain areas, we used the Isotropic Fractionator technique (Herculano‐Houzel and Lent 2005). This method consists of the homogenization of dissected paraformaldehyde‐fixed brain structures into an isotropic suspension of isolated nuclei that are counted in a hemocytometer for the determination of the total number of cells in the original dissected structure (assuming that all cells were collected and that all brain cells have one and only one nucleus). In a second step, a representative sample of the homogenate is subjected to immunocytochemistry to NeuN to establish what fraction of all DAPI+ nuclei also express NeuN and can thus be considered to be neuronal (Mullen et al. 1992; Gittins and Harrison 2004; Ngwenya et al. 2016; Swiegers et al. 2018; Imam et al. 2022; Matsushima et al. 2022).

Briefly, each sample was homogenized in a dissociation solution (1% Triton X‐100 in 40 mM sodium citrate) that helps to dissolve cell membranes, conserving the nuclear membranes, with a 2‐mL Wheaton Tenbroeck tissue grinder. Homogenates were collected with a glass Pasteur pipette and stained with the nuclear marker DAPI (4ʹ,6‐diamidino‐2‐phenylindole, 1 mg/mL). Density of nuclei was determined by manual counting of four aliquots of the stained suspension using a Neubauer Improved Brightline Chamber (Optik Labor) hemocytometer under a fluorescence microscope (Leica DMRA2) with x40 dry lens (0.75 numerical aperture). All counts produced coefficients of variation <0.10. Once the total number of nuclei was determined by multiplying the density of nuclei by the volume of each stained homogenate, an immunocytochemical protocol was applied to identify the fraction of neurons and nonneuronal cells in each brain region. Samples were centrifuged at 4°C for 5 min at 8000 rpm, and the supernatant was discarded. This step was repeated twice, and 500 µL of boric acid (0.2 M, pH 9.0) was added to each sample. Samples were then incubated for 45 min at 70°C in a water bath for epitope retrieval. The microtubes were centrifuged for 5 min at 8000 rpm, the supernatant was discarded, and 500 µL of PBS was added to the samples to wash the nuclei. Subsequently, 500 µL of blocking solution (0.30% Tween 20 and 1% BSA) was added, and samples were incubated at room temperature for 30 min at 350 rpm in a thermomixer (Eppendorf Thermomixer 5350). After blocking, the samples were centrifuged again at 4°C for 5 min at 8000 rpm, the supernatant was discarded, and the nuclei were incubated overnight (approximately 12–15 h) at 4°C with the labeled primary antibody Cy3 anti‐NeuN (rabbit polyclonal) Cy3 conjugate (RRID:AB_11204707, Millipore, Cat. No. ABN78C3) in a dilution of 1:100 in blocking solution under constant agitation in the thermomixer. Neuronal nuclear protein (NeuN) was used as a neuronal marker since it is expressed in the nuclei and perinuclear cytoplasm of neurons (Mullen et al. 1992). The following day, samples were centrifuged for 5 min at 8000 rpm, the supernatant was removed, and 500 µL of PBS (x1) was added to remove the excess of primary antibody. This step was repeated twice. A final volume of PBS (x1) was added to each sample, considering the amount of cells, to obtain cell densities suitable for cell counts in the brain regions: telencephalon and hindbrain, 100 µL; diencephalon + midbrain, 150 µL; and cerebellum and optic tectum, 200 µL.

### 2.5 Image Acquisition and Quantification

Since we use a fluorophore‐labeled primary antibody, our two‐step procedure for counting nuclei provides an internal control that shows a complete lack of visible red signal in DAPI+ nuclei prior to treatment with the labeled anti‐NeuN antibody. Thus, nuclei showing any detectable Cy3 signal can be considered immunoreactive for NeuN.

An in silico analysis was performed using a BLASTP search against the *Oreochromis* genus protein dataset available in NCBI to evaluate the specificity of the antibody in tilapia. The analysis focused on the antibody's binding site, located at the N‐terminal region of the protein, by comparing the N‐terminal sequence of the target protein in mouse with the corresponding region in the tilapia homolog. The BLAST results indicated that only the rbfox3 gene (NeuN) family in tilapia produced statistically significant alignments, with percent identities ranging from 85.71% to 95.24%, indicating that the antibody recognition site is highly conserved among species.

Quantification of the fraction of all DAPI^+^ nuclei that expressed NeuN immunoreactivity was performed by counting a minimum of 500 nuclei for each sample under a fluorescence microscope (Zeiss Imager/Apotome 2). In short, with a hydrophobic barrier pen, a rectangle was drawn in the middle of a coated glass slide (SuperFrost Plus), 5 µL of the cell suspension was added, and the preparation was coverslipped. With the Zeiss software, Zen 2 ‐ Blue edition software (V1.0 en. 5.7), the limits of the rectangle were set, and the images acquired automatically with a 20x dry lens (0.8 numerical aperture) in the DAPI channel and in the Cy3 (NeuN) channel. After image acquisition, DAPI and NeuN^+^ nuclei were quantified as a fraction of DAPI^+^ cells with Fiji software (V1.53c). Together with the Advanced Imaging Unit at the Instituto Gulbenkian de Ciência (IGC), we developed a macro that automatically counted the number of DAPI nuclei, the number of NeuN^+^ nuclei, and the overlap between the two. The macro was validated by comparing the number of cells counted by two human observers (M.C.T., G.M.M.) to the automated count in five samples of different brain regions, which yielded a correlation between the two methods of *R* ^2^ = 0.998 for DAPI and *R* ^2^ = 0.955 for NeuN‐positive nuclei. Additionally, we compared the distribution of nuclear sizes measured as Feret diameter in ImageJ and confirmed that NeuN+ and NeuN‐ groups had distinct nuclear diameters, as expected for neurons versus nonneuronal cells (Figure 1b). The number of neurons was determined for each brain area, and the number of other cells was determined by subtracting the number of neurons from the total number of nuclei determined previously. Densities were calculated as the ratio between the number of neurons and the mass of the structure (in mg).

### 2.6 Statistical Analysis

All statistical analyses were performed in JMP 16 (SAS, USA). Pairwise correlations between variables were calculated using a nonparametric Spearman rank correlation. Power law functions were obtained through least‐squares regression of natural log‐transformed data to a linear function. Two‐way ANOVA and *t*‐tests were also used to compare the means between the different treatments and investigate the interaction between the two independent variables (population density and age), followed by Tukey HSD post hoc tests to assess differences between each of the groups.

## 3 Results

### 3.1 Variation in Population Density Generates Variation in Body Size

**FIGURE 2:**
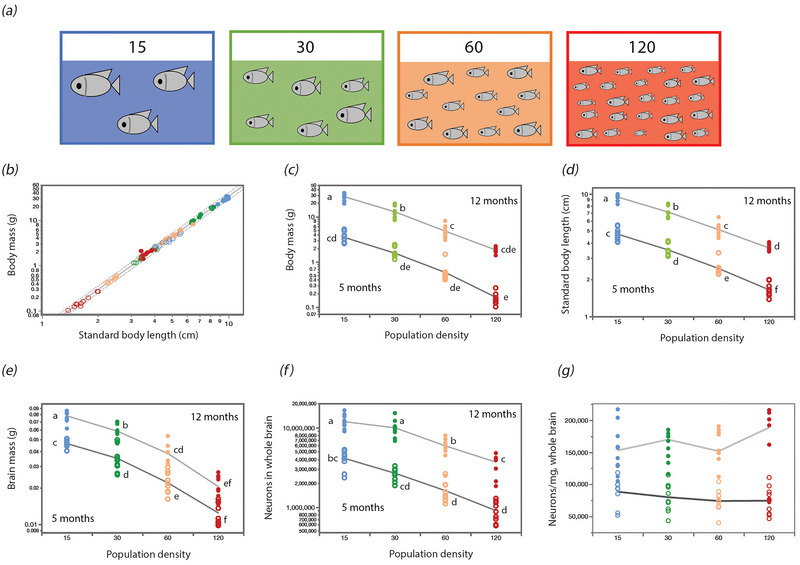
The combination of raising tilapia in four different population densities for either 5 or 12 months generated a continuum of body mass and body length. (a) Our experimental design of four groups, each with a different population density, indicated by the colors here and in all figures, from 15 fish/tank (blue, left) to 120 fish/tank (red, right). (b) Body mass (*M*_bd_) scales in our sample with approximately the cube of body length (*L*_bd_), as expected for isometric growth. The plotted function, which applies to all 80 individuals, is *M*_bd_ = e^−3.256 ± 0.027^ *L*_bd_^2.936 ± 0.018^ (*r* ^2^ = 0.997, *p* < 0.0001; dotted line shows the 95% prediction interval). (c–g) Fish raised in lower population densities have larger (c) body mass, (d) body length, (e) brain mass, (f) numbers of brain neurons (ANOVA across population densities within each age group, all *p* < 0.0001), but (g) unchanged neuronal densities (ANOVA, *p* = 0.45). In (g), no significant interaction effect was found for population density and age on neuronal density [*F*_(3, 70)_ = 1.57, *p* = 0.20] or for population density alone [*F*_(3, 70)_ = 1.57, *p* = 0.45]; only age had a significant effect (*F*_(1, 70)_ = 14.67, *p* = 0.0003). The lines connect the average values within the 10 individuals in each group of same age and population density. Unfilled symbols, 5‐month‐old tilapia; filled symbols, 12‐month‐old tilapia.

We analyzed a total of 80 individuals, 10 from each of four cohorts of different population density (Figure 2a) in either age group (5 or 12 months). Across the entire sample, individuals varied 7.2‐fold in standard body length (heretofore, “body length”), from 1.38 to 10.0 cm (younger adults, 1.38–5.56 cm; older adults, 3.39–10.0 cm), as shown in Figure 2b. Importantly, 37 individuals in either age group had overlapping body lengths in the range between 3.39 and 5.56 cm (Figure 2b), which allowed us to analyze any possible effects of slower growth rates on the relationship between the cellular composition of the brain and body size (length or mass).

The 80 animals varied 335‐fold in body mass, from 0.102 to 33.548 g (younger adults, 0.102–5.303 g; older adults, 1.398–33.548 g), with 30 individuals in either age group overlapping in body mass between 1.398 and 5.303 g (Figure 2b). As expected for isometric growth, we find that body mass scaled with very nearly the cube of body length (exponent, 2.936 ± 0.018; Figure 2b), and the smallest individuals raised for 12 months at highest population density overlapped in body mass and length with the largest individuals raised for 5 months, providing a continuum of body sizes in our sample (Figure 2b). Fish raised in lower population densities have larger body mass, body length, brain mass, and number of neurons (Figure 2c‐f ); however, neuronal density remains unchanged (Figure 2g).

### 3.2 Across Growth Conditions, Larger Animals Have Larger Brains, but Relative Brain Size Depends on Growth Rate

Raising the tilapia under conditions of increased population density markedly decreased body size (mass and length) at both 5 and 12 months, as shown in Figure 2c,d, with significant effects of population density and age, as well as a significant interaction between them (ANOVA, all *p* < 0.0001). At both ages, higher population densities also caused highly significant decreases both in brain mass (Figure 2e) and in total numbers of neurons in the brain (Figure 2f; ANOVA, all *p* < 0.0001). Only age (ANOVA, *p* = 0.0003) and not population density (ANOVA, *p* = 0.45) had a significant effect on neuronal density (Figure 2g). Interestingly, our manipulation produced an 8.8‐fold variation in brain mass (minimum, 0.0098 g; maximum, 0.0865 g; Figure 2e) and a much larger, 28.1‐fold variation in numbers of neurons in the whole brain (minimum, 584,447; maximum, 16,422,728 neurons; Figure 2f) across all 80 individuals.

**FIGURE 3:**
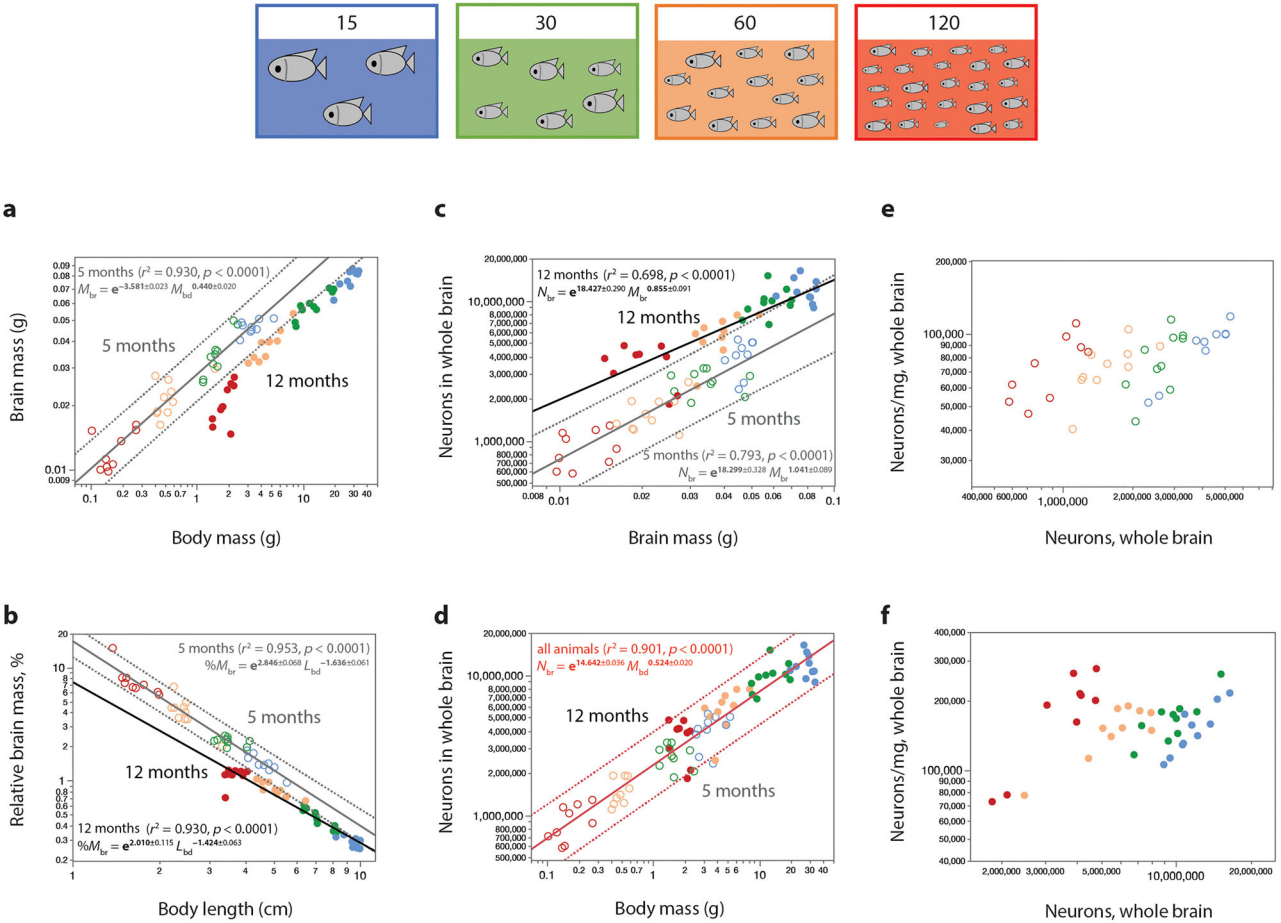
Larger animals, with larger body mass (*M*_bd_) and body length (*L*_bd_), have more brain neurons (*N*_br_), but their brain mass (*M*_br_) and neuronal density (*N*_br_/mg) depend on raising conditions. (a) Across animals raised to age 5 months (unfilled circles), brain mass scales with body mass (plotted in gray; dotted line shows the 95% prediction interval). At age 12 months, the function that applies is *M*_br_ = e^−4.122 ± 0.041^ *M*_bd_^0.494 ± 0.018^ (*r* ^2^ = 0.950, *p* < 0.0001, not plotted). Fish allowed to grow until age 12 months (filled circles) have higher body and brain masses compared to their siblings at 5 months but lower brain mass compared to their kin who reached a similar body mass already at 5 months. (b) Relative brain mass (%*M*_br_) decreases with increasing body length differently in animals at 5 and 12 months, with almost all data points for 12 months falling outside the 95% prediction interval for the scaling relationship that applies at 5 months. (c) In animals raised for 5 months, numbers of brain neurons scale with brain mass almost linearly; across siblings raised to 12 months, the function is also indistinguishable from linearity (plotted in black; 95% confidence interval omitted for clarity). (d) Regardless of the different scaling of brain mass with body mass across raising conditions, numbers of neurons in the whole brain vary as a single power function of body mass, plotted in red across all individuals aged 5 or 12 months. The power function for body length is *N*_br_ = e^12.950 ± 0.091^ *L*_bd_^1.529 ± 0.060^ (*r* ^2^ = 0.896, *p* < 0.0001, not plotted). (e, f) In agreement with the near‐linearity of the scaling relationships between brain mass and number of neurons in (c), neuronal density does not vary strongly with increasing numbers of neurons across all animals and population densities at 5 months (e: *r* ^2^ = 0.272, *p* = 0.0008) and 12 months of age (f: *r* ^2^ = 0.104, *p* = 0.0421). The color code indicates population density, according to the diagram. Unfilled symbols, 5‐month‐old tilapia; filled symbols, 12‐month‐old tilapia.

We next investigated whether larger animals had larger brains across all conditions. Figure 3a shows that across all animals raised to 5 months of age that reached different body masses by growing at different population densities, larger bodies come with larger brain mass (power function, *r* ^2^ = 0.930, *p* < 0.001). Interestingly, though at age 12 months animals had both larger bodies and brains compared to their siblings collected at age 5 months, the relationship between brain mass and body mass was not a simple extension of the power function that applied at 5 months (Figure 3a). Rather, animals that reached a given body mass at 12 months of age at high population densities of 60 or 120 fish/tank attained brain masses that were significantly smaller than those seen in younger siblings that reached a similar body mass in just 5 months at lower population densities of 15 or 30 fish/tank, respectively, falling outside the prediction interval (Figure 3a). For instance, animals of ∼3.5 cm standard length, which have ∼2 g body mass, have a brain twice as large for their body size (3% compared to less than 1.5% of body mass, *t*(18) = −15.26, *p* < 0.0001) when they reach that body size by growing rapidly in a low population density rather than by growing slowly in a high population density. (Figure 3b). This finding indicates that brain mass does *not* scale universally with body mass regardless of how a certain body mass was attained; rather, the relative size of the brain (which is the percentage of body mass occupied by the brain, not the residual brain mass after accounting for scaling with body mass) is markedly impacted by the growth rate of the animal (Figure 3b).

### 3.3 Brains of Similar Size Have Different Numbers of Neurons Depending on Growth Conditions

We find that animals with a given brain mass when raised for 12 months in higher population densities had more than twice as many neurons as their siblings that attained a similar brain mass at age 5 months by growing in lower population densities (Figure 3c). While this finding could be dismissed as a consequence of more neurogenesis accumulated over time, a simple effect of time cannot account for the converse finding of similar numbers of brain neurons in the small brains of 12‐month‐old animals raised at 120 fish/tank and in the much larger brains of 5‐month‐old animals raised at 15 fish/tank (Figure 3c). The interaction between how long it took each fish to produce a certain number of neurons—that is, the effect of growth conditions—and the brain size that it reaches is also captured on the much lower neuronal densities in the brains of fish at 5 months of age (Figure 3e) compared to the twofold higher densities in 12‐month‐old fish with similar numbers of neurons (Figure 3f). Importantly, while growing in different population densities starkly affects body mass, brain mass, and numbers of neurons in the brain (Figure 3a–d), there is no marked effect of population density on neuronal density within each age group (Figure 3e,f).

Strikingly, we find that the total number of neurons in the brain is a single function of both body mass and body length, across both ages and four population density conditions (Figure 3d), such that animals of a similar body size, regardless of how rapidly it was attained, and whatever the brain mass, have similar numbers of brain neurons, with about 90% of the variance in numbers of neurons explained by variation in body size (whether mass or length; Figure 3d).

### 3.4 Individual Brain Regions Show Similar Scaling Patterns That Depend on Growth Conditions

**FIGURE 4:**
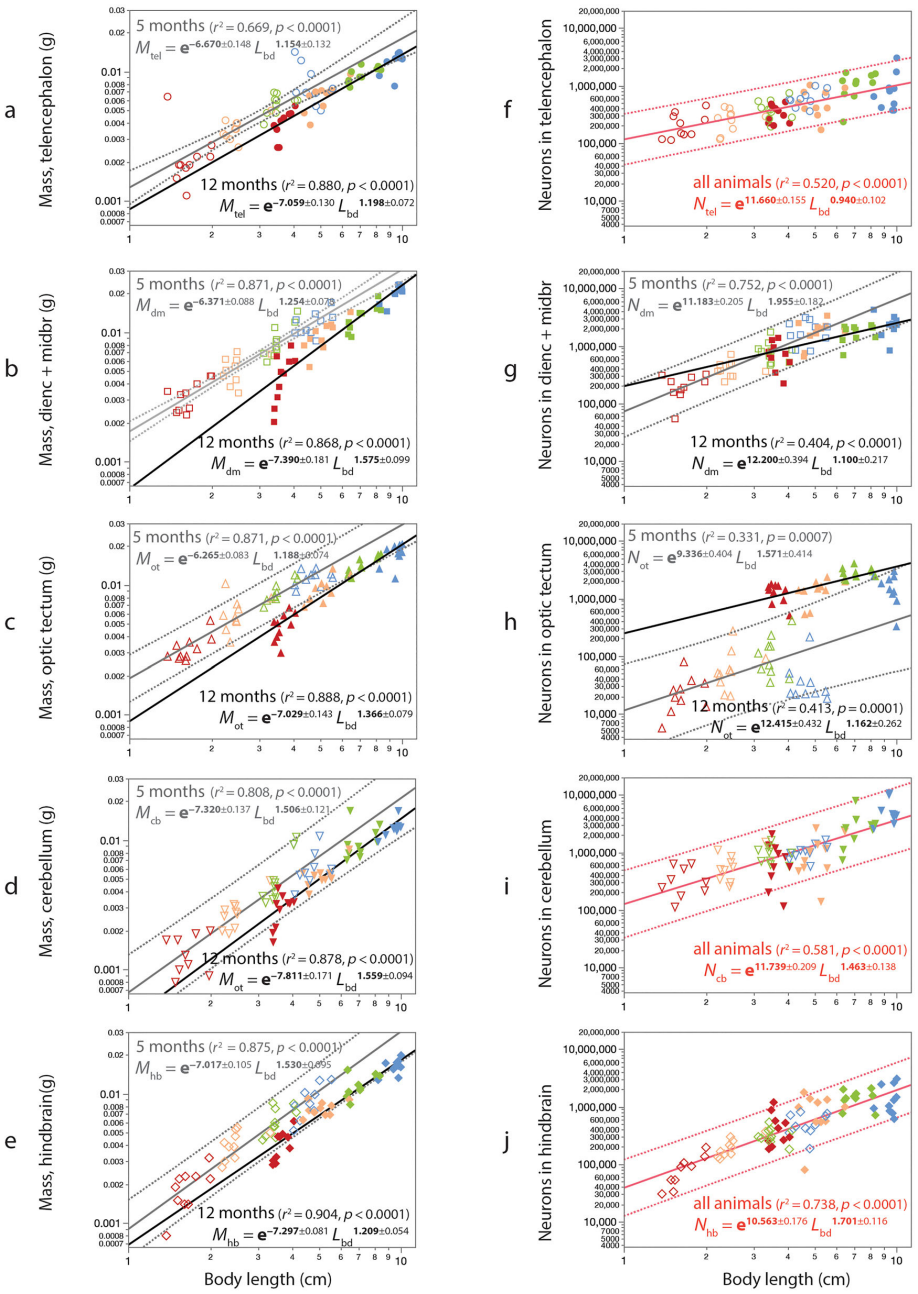
Raising conditions impact the scaling of the mass of most brain structures (*M*_str_), but larger animals have more neurons in the different brain structures (*N*_str_) regardless of raising conditions, with the exception of the optic tectum. Graphs show the mass (a–e) and numbers of neurons (f–j) of different brain structures plotted as a function of body length (*L*_bd_). Dotted lines indicate the 95% prediction interval for the power functions that apply at 5 months (12 months not shown for clarity). (a–e) Larger animals collected at both time points have larger mass in all brain structures than smaller animals of a similar age. However, for animals that have taken either 5 or 12 months to reach a same body length, brain structure mass in the slow‐growing animals is systematically below that expected for animals that had attained that body length in 5 months of growth. Graphs show (a) telencephalon; (b) diencephalon and midbrain; (c) optic tectum; (d) cerebellum; and (e) hindbrain. (f–j) Numbers of neurons scale uniformly with body length across all ages and conditions in the telencephalon (f), cerebellum (i), and hindbrain (j). In the combined diencephalon and midbrain (g), there are more neurons in the diencephalon of the largest fish raised for 5 months compared to the diencephalon of a fish that had attained a similar size in 12 months. Conversely, the optic tectum of fish of a certain body length has about 10 times more neurons after 12 months of growth compared to fish that reached a similar body length in just 5 months of growth (h). Functions plotted in (h) exclude the 10 animals raised under the lowest population density (blue), which are clear outliers. As in all figures, increasingly “hot” colors indicate higher population densities (blue, 15 animals; green, 30 animals; orange, 60 animals; red, 120 animals). Unfilled symbols, 5‐month‐old tilapia; filled symbols, 12‐month‐old tilapia.

Once we observed that larger animals have larger brain mass across the entire sample, we next examined whether this pattern extended to each of the five subdivisions of the tilapia brain: telencephalon, diencephalon/midbrain, optic tectum, cerebellum, and hindbrain. Figure 4 shows that in each of the two age groups, all brain structures have a larger mass in larger animals. However, for a given body length, the mass of each brain subdivision is smaller in animals that took 12 months to reach that body length (due to growth in higher population densities) compared to animals that reached that body length more rapidly, in just 5 months (due to growth in lower population densities). In other words, brain structure mass in the slow‐growing animals is systematically below the expected for animals that had attained that body length in 5 months of fast growth. Notice that the exponents relating brain structure mass and body length vary between 1.2 and 1.6, that is, significantly below the exponent of 3.0 expected for isometric growth with body length, but also consistently above the exponent of 1.0 expected if brain structure mass depended on body length.

**FIGURE 5:**
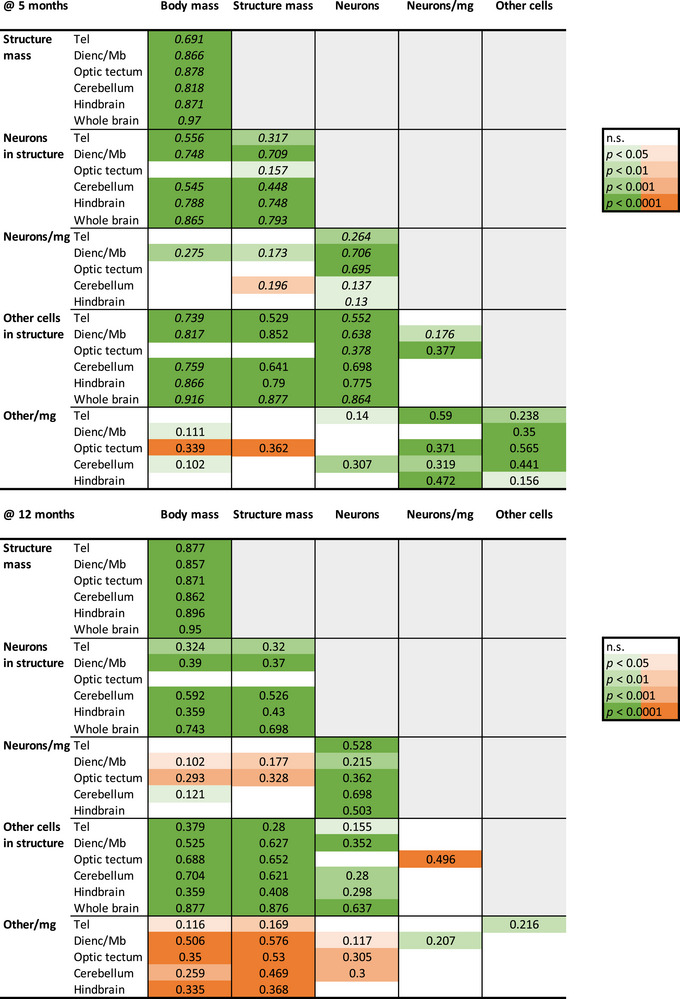
Across animals in all four population densities, larger body mass consistently scales with larger brain structure mass and numbers of neurons and of other cells within each of the two age groups. Neuronal density consistently scales positively with variation in numbers of neurons in each brain structure, but not with body or brain structure size. Strikingly, other cell density scales negatively in a consistent manner with body and structure mass, but only in the older cohort. The color code indicates the *p*‐value of the power function (green, positive exponent; orange, negative exponent) that relates each pair of variables in each brain structure (Tel, telencephalon; Dienc/Mb, diencephalon and midbrain), according to the color key. n.s., not significant.

As observed for the brain as a whole (Figure 3), we find that, while brain structure mass seems to be sensitive to growth conditions, the total number of neurons in the telencephalon, the cerebellum, and the hindbrain is a single function of body length across both ages and all four population density conditions (Figure 4), such that animals of a similar body length, regardless of how rapidly it was attained, have similar numbers of neurons in these brain structures. Thus, the effect of growth conditions on numbers of neurons and brain mass is not coupled to each other across most brain regions. The exception is the optic tectum, where animals that took longer to reach a certain body length systematically have ∼10 times more neurons than animals that reached that body length in just 5 months (Figure 4h). Additionally, at each time point, the largest animals have far fewer neurons in the optic tectum than expected for their body length. The pattern of scaling across the four populations within each age group is illustrated in Figure 5.

### 3.5 Within Each Cohort Raised at a Single Population Density, Larger Animals Have Larger Brain Structures but Not More Neurons

**FIGURE 6:**
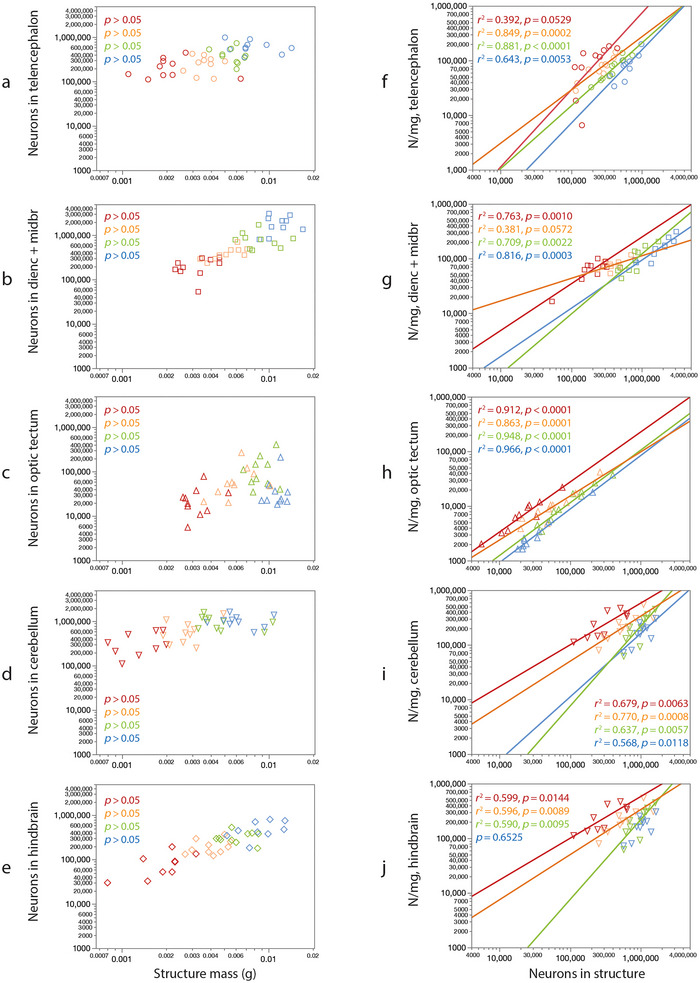
Individual variation in brain structure mass, number of neurons, and neuronal density at 5 months. (a–e) Fish raised in higher population densities have smaller brain structure mass and fewer neurons that are, however, not correlated across individuals in a same tank. (f–j) Within each condition, larger numbers of neurons in a brain structure occur with higher neuronal densities across individuals. Data points show individual fish; colors indicate population density in each tank (blue, 15; green, 30; orange, 60; red, 120). Animals with larger brain structures within each condition do not have more neurons in that structure. Values of *r* ^2^ and *p* refer to the significant power functions that apply to each scaling relationship, mapped in Figure 8.

**FIGURE 7:**
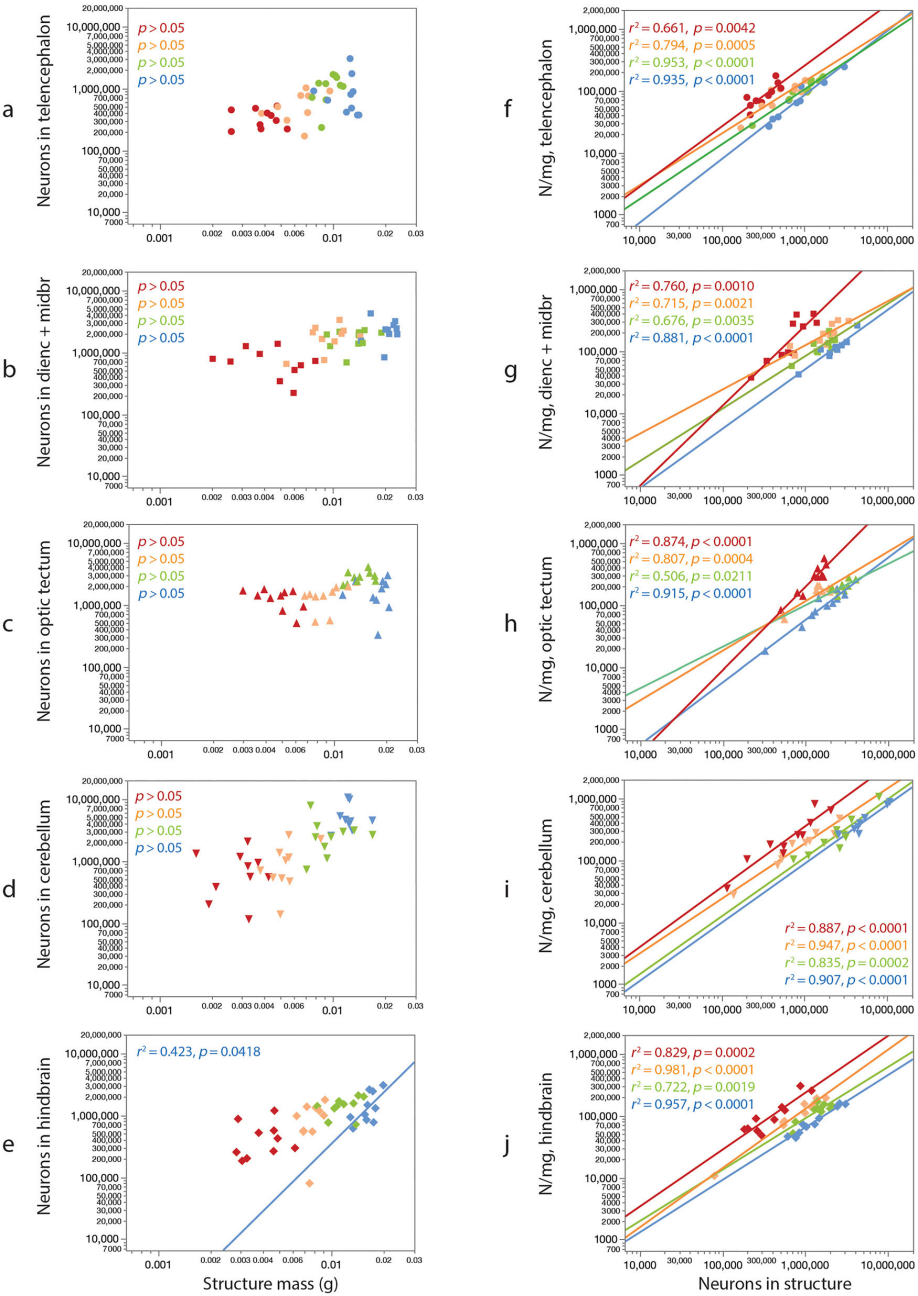
Individual variation in brain structure mass, number of neurons, and neuronal density at 12 months. (a–e) Tilapia with larger brain structures have larger brains and more neurons across cohorts raised at different population densities, but not within cohorts raised at a same population density. With the exception of the hindbrain at 15 fish/tank, animals with larger brain structures within each cohort do not have more neurons in that structure. (f–j) Within each cohort, larger numbers of neurons in a brain structure occur with higher neuronal densities across individuals. Data points show individual fish; colors indicate population density in each tank (blue, 15; green, 30; orange, 60; red, 120). Values of *r* ^2^ and *p* refer to the significant power functions that apply to each scaling relationship, mapped in Figure 8.

**FIGURE 8:**
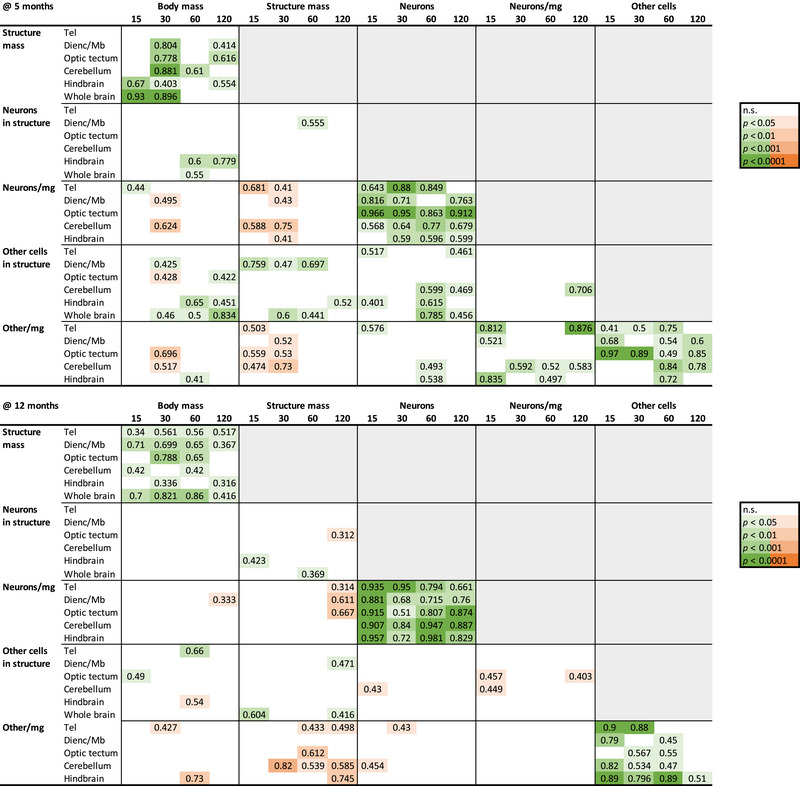
Within each cohort and age group, body mass and brain structure mass scale consistently together across individuals, and so do numbers of neurons and neuronal density, as well as numbers of other cells and other cell density, but larger body or brain size does not correlate with more neurons or other cells or larger or lower densities of either cell type. The color code indicates the *p*‐value of the power function (green, positive exponent; orange, negative exponent) that relates each pair of variables in each brain structure (Tel, telencephalon; Dienc/Mb, diencephalon and midbrain), according to the color key. n.s., not significant.

Importantly, the scaling of brain structure mass or number of neurons with body length that applies across population densities does not hold *within* each cohort raised at a single population density, at each age (Figures 3 and 4). In each of these, we find that larger structures, with rare exceptions, do *not* have more neurons, neither at 5 months (Figure 6, left column) nor at 12 months (Figure 7, left column). The exception is the increased numbers of hindbrain neurons with increased hindbrain mass in 12‐month‐old fish reared at 15 fish/tank (Figure 7e). The lack of significant correlation within each cohort cannot be attributed to a small *n* or a low amplitude of variation, as we find that larger animals within a cohort do have larger brain structures (Figure 4, scaling relationships summarized in Figure 8), and brain structures with more neurons have higher neuronal densities, always with a power exponent identical to unity (Figures 6 and 7, right column), which mathematically can only result from individual variation in numbers of neurons in a manner that is independent of variation in structure mass within each cohort.

### 3.6 Nonneuronal Cell Variation Accompanies Neuronal Variation

**FIGURE 9:**
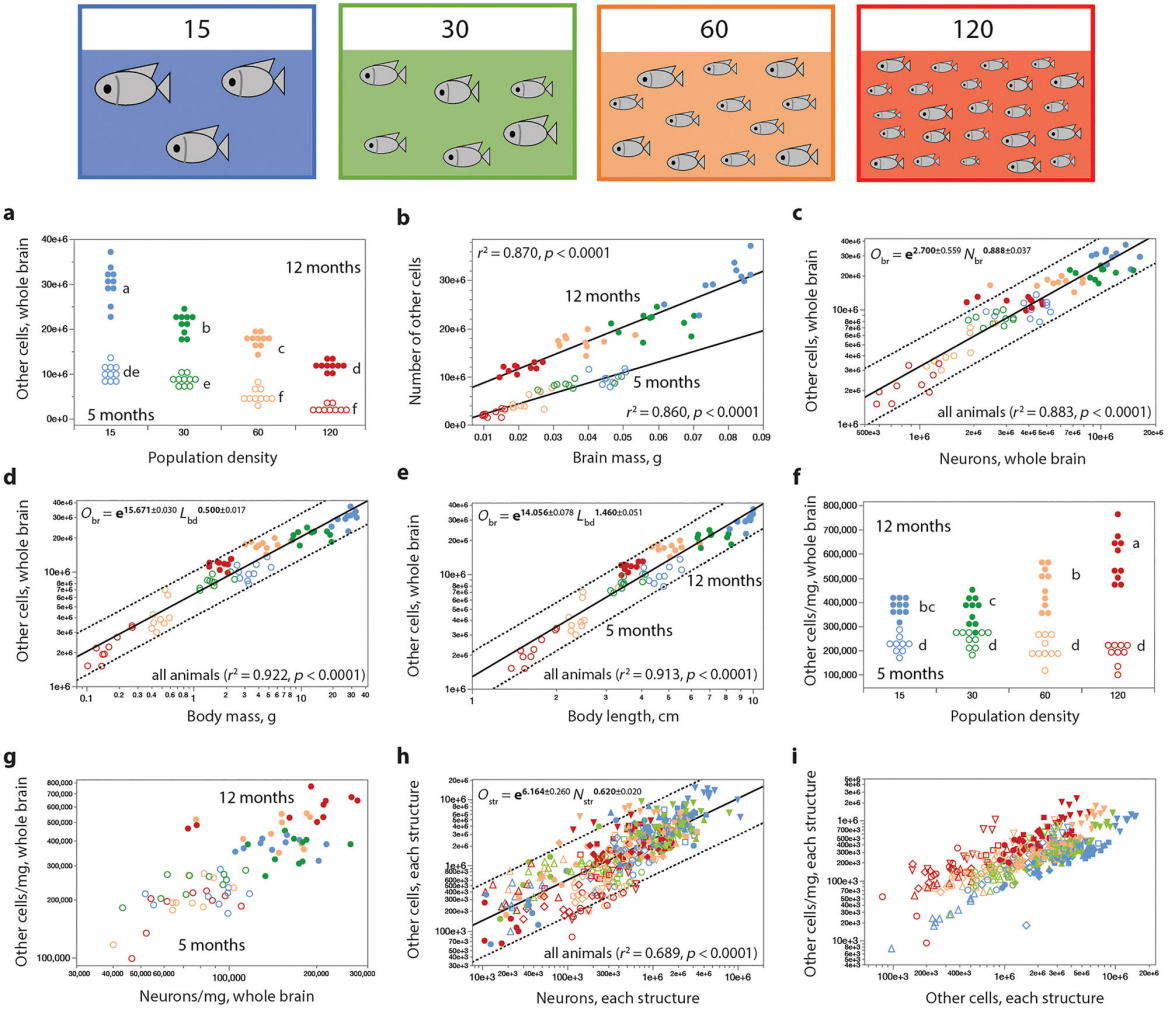
Numbers of other cells vary together with numbers of neurons across cohorts and individuals. (a) Fish raised in lower population densities have larger numbers of other (nonneuronal) cells in the brain. (b) Across all individuals in each age group, the total number of other cells in the brain varies linearly with brain mass (linear functions plotted; *r* ^2^ and *p*‐values are indicated in the graph). (c) Across all animals, the number of other cells in the brain scales with the number of neurons according to the function plotted. The distribution can also be fitted with a linear function of slope 2.002 ± 0.112 (*r* ^2^ = 0.806, *p* < 0.0001; not plotted). Power functions that apply to each age group have exponents 0.952 ± 0.062 (at 5 months; *r* ^2^ = 0.864, *p* < 0.0001) and 0.519 ± 0.064 (at 12 months; *r* ^2^ = 0.637, *p* < 0.0001; not plotted). (d) Regardless of the different scaling of brain mass with body mass across raising conditions, numbers of other cells in the whole brain vary as a single power function of body mass, like neurons do. (e) Similarly, numbers of other cells in the whole brain vary as a single power function of standard body length, like neurons do. (f) Other cell density does not vary significantly with population densities at 5 months, although it is significantly higher in 12‐month‐old animals raised in the highest population densities. We found an interaction effect of population density and age (*F*_(3, 70)_ = 21.30, *p* < 0.0001) and significant main effects for both factors (*F*_(3, 70)_ = 8.95, *p* < 0.0001 and *F*_(1, 70)_ = 39.46, *p* < 0.0001, respectively). (g) While whole‐brain other cell density appears to vary together with neuronal cell density across the entire dataset, there is no systematic correlation found for any brain structure within an age group (Figure 5) or within a cohort (Figure 8). (h) Across all animals, ages, and brain structures, the number of other cells in a structure is a power function of the number of neurons in the structure, as indicated in the graph, although this is not true within each cohort (Figure 8). (i) For each brain structure, the more the other cells composing the structure, the higher the density of other cells, as summarized in Figure 8. For each function plotted, the dotted line shows the 95% prediction interval. The color code indicates population density, according to the diagram. Unfilled symbols, 5‐month‐old tilapia; filled symbols, 12‐month‐old tilapia. Different letters indicate differences between treatments, *p* > 0.05.

In each of the two age groups, animals raised in progressively higher population densities also had progressively fewer other (nonneuronal) cells (Figure 9a), which ranged from a minimum of 1.503 million to 37.142 million cells across all individuals. ANOVA analysis showed significant effects for population density and age as well as for their interaction (all *p* < 0.0001). The number of other cells increases linearly together with brain mass (Figure 9b), but as distinct functions for each age, such that animals raised for 12 months in higher population densities had more than twice as many other cells as their siblings that had grown to a similar brain mass in 5 months of growth in lower population densities. For example, 12‐month‐old fish raised at 120 fish/tank have a similar brain mass as 5‐month‐old fish raised at 60 fish/tank (*t*(18) = −0.731, *p* = 0.473) but significantly more nonneuronal cells (*t*(18) = 11.364, *p* < 0.0001). Again, the finding of approximately similar numbers of other cells in the small brains of 12‐month‐old animals raised in high population densities and in the much larger brains of 5‐month‐old animals raised in low population densities dismisses a simple accumulation of more cells generated over time as the explanation for the larger numbers of other cells in the brains of 12‐month‐old animals compared to 5‐month‐old animals (Figure 9b) in favor of generation of numbers of cells according to population density‐dependent opportunities for growth.

We find that total numbers of neurons and other cells vary consistently together, and nearly linearly, across all individuals in our dataset, both within each age group and across them (Figures 5 and 9c), with a linear fit across all 80 individuals that has a slope of 2.00 other cells for every neuron in the brain. Accordingly, we find that the total number of other cells in the brain varies with body mass (Figures 5 and 9d) and with standard body length (Figure 9e) raised to exponents of 0.500 ± 0.017 and 1.460 ± 0.051, similar to the exponents that applied to total numbers of brain neurons. The one distinction we find in the variation of other cells compared to neurons is that other cell densities are significantly elevated in the 12‐month‐old animals in all population densities compared to their 5‐month‐old siblings (ANOVA for population density and age, as well as for their interaction, *p* < 0.0001; Figure 9f). The small variation in whole‐brain neuronal density across animals is not accompanied by significant variation in other cell density within each age group (Figure 9g; *p* > 0.0001).

In each brain structure, numbers of other cells and neurons also vary consistently together across all cohorts within each age group and, strikingly, in an overlapping manner across all structures (Figures 5 and 9h). As found for neurons, we find that those structures with more other cells have higher other cell densities, although across the individuals raised in the various population densities, this relationship is only consistent in the 5‐month‐old animals (Figures 5 and 9i). At 12 months, other cell density decreases with increasing body and brain size (Figure 5).

Again, as found for neurons, we find that within each cohort of individuals raised in a given population density for either 5 or 12 months, the only consistent correlation is between the number of other cells in each brain structure and the density of other cells in that brain structure (Figure 8). These correlations are shown in Figure 9i. Importantly, there is no consistent covariation in numbers of neurons and numbers of other cells in a brain structure across individuals raised in a given population density for either 5 or 12 months (Figure 8), as can be seen in Figure 9h.

## 4 Discussion

Here, we show that changing population density is a powerful manipulation to modify body mass, brain structure mass, and numbers of neurons and other cells in the different brain structures. We find that within each cohort of animals raised under a given population density, larger animals have larger brains that do not have more neurons, while animals with more neurons have higher densities of neurons. These findings confirm and extend to fish the pattern of brain scaling found across individuals within a same species, both for rodents (Herculano‐Houzel et al. 2015) and for birds (Cunha et al. 2022). The pattern that applies within each cohort is, however, in striking contrast to what we find across cohorts of fish raised in different population densities: that larger animals have larger brains with up to 20‐fold more neurons and other cells whose density either decreases or remains constant, but does not increase, as brain structure size and/or number of cells increases. Remarkably, this latter pattern of scaling across growth conditions mimics the by now well‐documented patterns of scaling of the cellular composition of the brain across species (Herculano‐Houzel et al. 2014; Herculano‐Houzel and Rothman 2022), even though the individuals in question were all from the same stock. To reconcile these findings, we propose that the contrasting scaling of brain diversity that we report within cohorts (raised as a single population of a certain population density and thus growth conditions) and across cohorts (raised at different population densities and growth conditions) can be explained by brain plasticity in response to changing environmental conditions, that is, under different opportunities for growth, which scales up or down the intrinsic variation in developmental mechanisms that govern brain size and number of brain neurons. If environmental opportunities are maintained over a number of generations, the sustained brain plasticity may underlie the evolutionary development of brain diversity across species and clades. We detail this proposition below.

Population density dependence is characterized by a decline in growth rates as population size increases, which influences body size and life‐history trajectories, presumably due to intensified competition for limited resources (Holm et al. 1990; Saether 2024). While environmental pressure may shape evolutionary strategies by favoring life‐history traits that improve survival and reproductive success under crowded conditions, our experimental setup specifically eliminated selective effects and adaptation; rather, we must only consider immediate effects of rearing conditions on brain and body growth. While a few studies have established that rearing population density impacts brain size (relative, absolute, and residual, respectively) of different parts of the brain (Fisher et al. 2015; Näslund et al. 2016, 2019; Triki et al. 2019), we are the first to demonstrate that population density also affects the numbers of neurons that compose the different brain structures, in a manner that depends on growth rate.

In our experiment, we used four levels of population density to generate a continuous range of body sizes in order to study intraspecific differences in brain plasticity. Higher population densities are known to bring about heightened stress responses that influence growth rate (Swain et al. 2022). In fish, it is well established that stressful stimuli can trigger neurohormonal responses in the hypothalamic–pituitary–interrenal (HPI) axis, as well as cortisol release. Cortisol levels increase significantly with stocking density (Swain et al. 2022), and elevated cortisol levels achieved in salmonids with cortisol implants significantly reduced growth rates (Vargas‐Chacoff et al. 2021). Elevated cortisol levels can suppress growth hormone (GH) and insulin‐like growth factors (IGF‐I) through direct inhibition, induction of GH resistance, and upregulation of inhibitory IGF‐binding proteins (IGFBPs), resulting in decreased feed intake and growth (Canosa and Bertucci 2023). Additionally, social environmental stress also impacts neurogenesis through decreased numbers of newborn cells (Sørensen et al. 2012; Tea et al. 2019; Teles et al. 2023; Maruska et al. 2012). Increased population densities may therefore also impact brain development and growth by imposing a heightened social stress on individuals. It seems that one way or another, then, increased population densities lead to increased stress responses.

We thus suggest that the progressively smaller numbers of neurons that we find in fish raised in progressively higher population densities arise from stress‐mediated decreased neurogenesis. One might argue instead that the smaller numbers of neurons in these circumstances are rather a result of the effect of stress to slow growth and decrease body size, which would then lead to an associated number of neurons. However, our finding that numbers of neurons are unrelated to body size within a cohort implies that reduced body size on its own is not sufficient to cause reduced numbers of neurons. Our finding of concerted variations in numbers of cells across all brain structures in response to changing population density is strongly suggestive of global effects of growth conditions on not only cell differentiation but also cell proliferation, consistent with system‐wide hormonal effects. Additionally, the finding that numbers of both neurons and other cells are larger in animals raised under lower population density conditions indicates that increased numbers of neurons are not simply the result of conversion of undifferentiated cells or numbers of other cells would decrease as numbers of neurons increase in a trade‐off, which does not happen.

Importantly, continued neurogenesis in fish is not a simple response to continued growth, as zebrafish have continued neurogenesis (Zupanc 2021) but not indeterminate growth (Biga and Goetz 2006), which indicates that body (and brain) growth and neurogenesis are regulated through different mechanisms, even if they can be affected by the same variables. Additionally, our data provide evidence that brain and body size can be uncoupled by changing growth conditions: under more favorable conditions (lower population density), a given brain mass is achieved faster (in 5 months, instead of 12 months) and occupies a body that is comparatively smaller. The resulting larger relative brain mass of animals of a given length that grew under more favorable conditions for growth (i.e., lower population density) is consistent with the priority that brain growth takes over the growth of all other organs of the body in vertebrates (Heldstab et al. 2022). Strikingly, while relative brain size and the relationship between numbers of neurons and brain mass are sensitive to growth conditions, the relationship between numbers of neurons and body mass is shared across our entire dataset (though not within each cohort raised as a single population of a given density; see below). This relationship applies to all brain structures with the single exception of the optic tectum, whose distinct sensitivity to the different growth conditions imposed by different population densities resulting in a lack of overall correlation between body mass and number of tectal neurons is evidence that its postnatal development follows a different trajectory from other brain structures. It will be interesting to pursue the mechanisms and implications of this distinction of the optic tectum, given its role as a central hub for visuomotor transformations (Basso and May 2017) that mediates orienting movements toward stimuli (approach) and initiates evasive responses to threats (escape) (Suzuki et al. 2019, Helmbrecht et al. 2018). The complexity and very large numbers of neurons of this structure may be constrained by developmental processes to lag behind other brain structures as a whole, explaining the lack of continuity between 5‐month‐old and 12‐month‐old individuals in the relationship between body mass and numbers of tectal neurons (Figure 4h). This would be consistent with the finding that at the lowest population density, the most conducive to growth, large body sizes are achieved without a matching number of neurons in the optic tectum having been reached yet, both at 5 and at 12 months of age (Figure 4h). Alternatively, visual understimulation in the lowest density cohort may have impaired the production of neurons, since tectum size and organization are known to vary widely based on ecological niche and reliance on vision (Yopak and Lisney 2012). This could result in the inverted U‐shaped type curve observed in Figure 4h, which appears more pronounced in younger animals.

The seemingly universal correlation we find between numbers of neurons and body mass or length across our entire dataset is in line with the numerical matching hypothesis that posits that peripheral elements, such as muscle fibers or sensory receptor cells, and central elements in the brain, responsible for motor control of those muscles or the processing of sensory input, scale together (Zupanc 1999, 2021), presumably due to elimination of those neurons that fail to find a target to innervate. Examples of the numerical matching hypothesis include motoneuron growth in fish and chick embryos in response to muscle development, matching between retinal cells and tectal neurons in fish, and ongoing neurogenesis in the mammalian olfactory system to align sensory input with central processing capacity (for review, see Zupanc [2021]). Alternatively, seemingly matching numbers of neurons and body mass might result simply from both variables responding to a shared environmental factor, as we propose below.

On the other hand, the complete lack of correlation between body (or brain) size and number of neurons *within* each cohort of animals raised under a given population density contradicts the numerical matching hypothesis. However, the lack of correlation between body (or brain) size and number of neurons within each cohort should come as no surprise given previous findings that brain size and number of brain neurons are regulated by different genetic mechanisms in mice (Williams et al. 1998; Rosen and Williams 2001), just like brain size and body are regulated by independent genetic loci in chickens (Henriksen et al. 2016).

The contrasting patterns of neuronal, brain, and body scaling within cohorts (where all individuals grew under shared environmental conditions) and across cohorts (where environmental conditions were different by design) in our study can be reconciled in the following model, which we propose to account for the origins of diversity in brain scaling. Importantly, the model that we propose applies even in the absence of genetic mutations and selection. Our model is based on coupled reaction norms across traits, where a “reaction norm” refers to the range of phenotypes that a trait can produce across different environmental conditions, in a manner that is proportional to the environmental change (Aubin‐Horth and Renn 2009).

**FIGURE 10:**
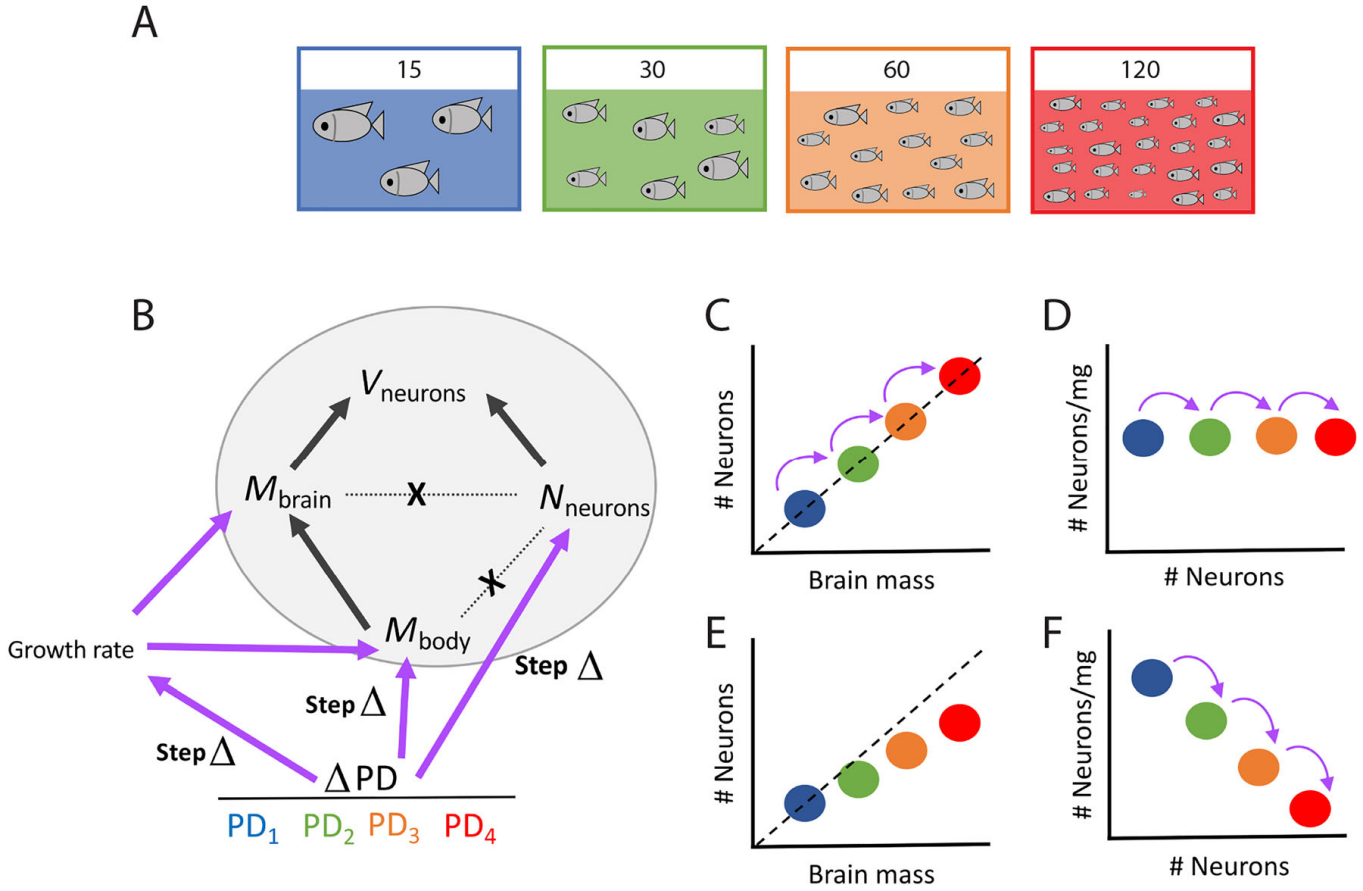
Proposed model of independent individual variation within a cohort in numbers of neurons on the one hand, and body and brain mass on the other, but coordinated step changes in both when environmental conditions change for whole cohorts. (a) Four groups of fish raised in increasing population densities PD_1_–PD_4_, indicated by the colors. (b) Numbers of brain neurons and brain mass vary independently across individuals in a cohort (the gray ellipse represents the variation within a cohort; the X indicates no correlation). Larger animals (*M*_body_) have larger brain mass (*M*_brain_), but not more neurons (*N*_neurons_), and thus no correlation between numbers of neurons and brain mass. As a result, those animals with more neurons will have smaller neurons (*v*_neurons_), leading to higher neuronal densities. (c–f) Changing the environmental conditions (different colors, as in b) in which the entire cohort develops causes coordinated step changes (indicated by the lavender arrows, also in b) in both body mass and brain mass and in numbers of brain neurons. When numbers of neurons and brain mass undergo step changes of similar magnitude (c), neuronal density remains unchanged across cohorts (d), as found across species of primates, songbirds, and parrots. On the other hand, when step changes cause a larger increase in brain mass than in numbers of brain neurons (e), for example, through changed growth rates, the result is dropping neuronal densities across cohorts (f), as found across most species of mammals. Repetition of the process over time leads to allometric scaling across species in a clade (within c and d, or e and f) that may also differ across clades (between c and d and between e and f).

Within each cohort, brain mass and body mass vary in coordination across individuals, but numbers of neurons vary independently from both (Figure 10b, gray ellipse). We propose that macroenvironmental step changes in growth opportunities, such as those introduced by the different population densities in our experiment, result in cohorts with separately changed brain and body mass, on the one hand, and numbers of neurons, on the other, due to their different reaction norms (Figure 10, lavender arrows). In the case that an environmental change at the cohort level results in *similar* degrees of change in brain size and number of brain neurons, then neuronal densities will remain unchanged across cohorts, as with the tilapia growing in different population densities in our experiment (Figure 10c,d). If the new macroenvironmental conditions are *sustained* over multiple generations, the result would be a new cohort that differs in body and brain size and number of neurons from the original cohort, like different *species* are found to do. We propose that such sustained, similar step changes in body and brain size and numbers of neurons occurring repeatedly across cohorts that were separated long enough that they became different species could explain brain scaling across species of primates (Herculano‐Houzel et al. 2007; Gabi et al. 2010) and songbirds and parrots (Olkowicz et al. 2016). In comparison, given a different genetic background, step changes in macroenvironmental conditions and opportunities for growth that caused a *faster* gain in brain mass than in numbers of neurons would generate cohorts (and possibly species, if sustained) that had more neurons accompanied by *lower* neuronal densities (Figure 10e,f)—as is the case across the species of mammalian and sauropsid clades, including rodents (Herculano‐Houzel et al. 2014; Kverková et al. 2022). At the same time, within each cohort, scaling relationships would remain dependent on stochastic, microenvironmental, epigenetic, and genetic factors, the latter of which are known to regulate brain size and numbers of neurons independently, as reviewed above.

A central predictive feature of our model is that the correlations that apply *across* cohorts are not an extension of the pattern of variation that applies *within* each group of animals because the former are the result of step changes in growth opportunities, while the latter remain the result of independent individual variation in the mechanisms that regulate numbers of neurons, body size, and brain size (Figure 10). The source of individual variation may be genetic, as reviewed above, but it may also be epigenetic or microenvironmental, for example, due to effects of social rank (Oliveira and Almada 1996) on either number of neurons or body or brain size. Because individual variation in numbers of brain neurons is not correlated with variation in brain size within a cohort, the consequence is that any increases in numbers of neurons result in linear increases in neuronal density in brain structures across individuals (Figure 10), as in our tilapia, and as reported across individual mice (Herculano‐Houzel et al. 2015) and chickens (Cunha et al. 2022). Thus, our model, illustrated in Figure 10, finally reconciles the apparent contradiction between intra‐ and interspecific scaling not only in brain size, numbers of neurons, and neuronal density but also between body and brain size (Armstrong 1990).

Our model of evolution of brain diversity through step changes in opportunities thus predicts that numbers of neurons and brain size (or body size) will appear correlated when both respond together, but in possibly different amounts, to environmental changes, even as these variables are not fundamentally correlated with each other. Similarly, we posit that there is not a single fundamental relationship between brain and body size, but rather one that depends on how the growth rates of brain and body are affected by step changes in environmental opportunities for growth. Acknowledging the lack of these fundamental universal correlations is transformative for understanding the mechanisms of origin of brain diversity because it implies that brain size is not a result of numbers of neurons, nor is the number of neurons that populate a brain a direct result of brain size, or brain size a direct result of body size. What is the missing link between changes in one and changes in the other? We suspect that one key factor is the balance between energy supply and energy use that underlies both growth rates and the largest sustainable brain size within a body, as well as the largest body size sustainable within the maternal or egg environment. We have recently found that adult brain economy is limited by its capillary density combined with rate of blood flow and not demand‐based as if energy supply were never a constraint (Herculano‐Houzel and Rothman 2022). Thus, changes in energy supply to the brain during early development brought about by changing environmental opportunities might affect, through different mechanisms, both the size of the brain that can be sustained and the numbers of neurons that can be generated (Iwata et al. 2023) and continuously supported. Importantly, it was recently discovered that mitochondrial function is inherited epigenetically from the mother (Lee et al. 2023), which, added to a sustained step change in environmental conditions, would also promote the continuity of the relationship between body size, brain size, and numbers of brain neurons across generations.

We thus speculate that the clade‐specific allometric scaling of interspecific variation in body and brain size and cellular composition results from the maintenance of shared reaction norms by related species across step changes in sustained macroenvironmental conditions, as depicted in Figure 10c–f, most likely due to inherited genomic and epigenomic traits. In turn, any genomic variation that caused changes in the reaction norms of changing environmental opportunities for growth would result in cohorts that scale in body size, brain size, numbers of neurons, and neuronal density according to a seemingly new scaling law—which, in retrospect, and over evolutionary time, will characterize a new clade.

The step changes in brain and body size and numbers of neurons that result from changing opportunities for development and growth are a form of phenotypic plasticity. If the conditions are maintained, these new traits (the changed body and brain size and number of neurons) should persist across generations, during which the accumulation of genetic drift might suffice to create incompatibility of its descendants with other descendants of the original allopatric cohorts. The result will be two cohorts that not only differ markedly in brain size, body size, and numbers of brain neurons but also have become reproductively isolated and can now be referred to as different species. We thus propose that new species, initially characterized by their distinct body and brain size, might arise quite simply through step changes in environmental opportunities for growth, as a natural consequence of brain phenotypic plasticity.

## Author Contributions

Conceptualization: Suzana Herculano‐Houzel and Rui F. Oliveira. Methodology: Magda C. Teles, Suzana Herculano‐Houzel, and Rui F. Oliveira. Investigation: Magda C. Teles and Gonçalo M. Melo. Visualization: Magda C. Teles and Suzana Herculano‐Houzel. Funding acquisition: Magda C. Teles and Rui F. Oliveira. Project administration: Rui F. Oliveira. Supervision: Magda C. Teles, Suzana Herculano‐Houzel, and Rui F. Oliveira. Original writing, review, and editing: Magda C. Teles, Suzana Herculano‐Houzel, and Rui F. Oliveira.

## Ethics Statement

The experimental work presented in this manuscript did not involve any experimental manipulation or animal procedures. As we explain in the methods section, the fish were raised in regular housing conditions, varying only the density of fish in each tank, and the biological samples were collected post‐mortem. Therefore, according to the National (DL 113/2013) and European (Directive 2010/63/EU) legislation, no ethical permit is required. Nevertheless, we send in attachment a declaration of the coordinator of the Animal Welfare and Ethics Body (ORBEA) of the ISPA‐Instituto Universitário, where this work was conducted.

## Conflicts of Interest

Suzana Herculano‐Houzel and Rui F. Oliveira, the corresponding authors of this article, are, respectively, the Editor‐in‐Chief and an Associate Editor of this journal. They were excluded from the peer‐review process and all editorial decisions related to the acceptance and publication of this article. Peer‐review was handled independently by JCN/CNE Journal editorial office and Dr. Jorge Mpodozis as Editor to minimize bias.

## Peer Review

The peer review history for this article is available at https://publons.com/publon/10.1002/cne.70090.

## Data Availability

The data that support the findings of this study are available from the corresponding author upon reasonable request.
